# From ageing clocks to human digital twins in personalising healthcare through biological age analysis

**DOI:** 10.1038/s41746-025-01911-9

**Published:** 2025-08-21

**Authors:** Murih Pusparum, Olivier Thas, Stephan Beck, Simone Ecker, Gökhan Ertaylan

**Affiliations:** 1 Environmental Intelligence, Flemish Institute for Technological Research (VITO), Mol, Belgium; 2 Data Science Institute, Hasselt University, Hasselt, Belgium; 3 Department of Applied Mathematics, Computer Science and Statistics, Ghent University, Ghent, Belgium; 4 National Institute for Applied Statistics Research Australia (NIASRA), University of Wollongong, Wollongong, NSW Australia; 5 UCL Cancer Institute, University College London, London, UK

**Keywords:** Personalized medicine, Epigenetics, Proteomics, Computational models

## Abstract

Age is the most important risk factor for the majority human diseases, leading to the exploration of innovative approaches, including the development of predictors to estimate biological age (BA). These predictors offer promising insights into the ageing process and age-related diseases. With real-time, multi-modal data streams and continuous patient monitoring, these BA can also inform the construction of ‘human digital twins’, quantifying how age-related changes impact health trajectories. This study highlights the significance of BA within a deeply phenotyped longitudinal cohort, using omics-based approaches alongside gold-standard clinical risk predictors. BA and health traits predictions were computed from 29 epigenetics, 4 clinical-biochemistry, 2 proteomics, and 3 metabolomics clocks. The study reveals that ageing is different between individuals but relatively stable within individuals. We suggest that BA should be considered crucial biomarkers complementing routine clinical tests. Regular updates of BA predictions within digital twin frameworks can also help guiding individualised treatment plans.

## Introduction

Age is the most important shared risk factor for the majority of human diseases. Hence, there are strong efforts towards attenuating ageing-related disease risk via lifestyle, pharmacological, and clinical interventions to slow or reverse ageing. ‘Chronological age’ (CA), defined by the time passed since an individual’s birth, falls short of reflecting interindividual and environmental differences acting upon a biological system during this period. A critical prerequisite in the endeavour to improve healthy ageing is to quantify an individual’s ‘wellness,’ which covers not only the absence of sickness but also their resilience to future disease, general satisfaction with their health, and having sufficient energy levels for activities that enrich one’s life. While a variety of signals related to individual health and well-being can be collected, validating their contribution to clinically relevant outcomes remains an open issue. The hallmarks of ageing include genomic instability, telomere attrition, epigenetic alterations, loss of proteostasis, disabled macroautophagy, deregulated nutrient-sensing, mitochondrial dysfunction, cellular senescence, stem cell exhaustion, altered intercellular communication, chronic inflammation and dysbiosis^1^. Each hallmark contributes to the ageing process. The major challenge is to dissect the interconnectedness between these hallmarks and their relative contributions to ageing^1^.

To achieve significant progress in the wellness and longevity area to improve human health span, we need to develop tools and methodologies for standard collection, harmonisation, analysis, integration, and interpretation of this information at the individual level. A variety of biological age (BA) predictors have been proposed. They share the common approach of using large cross-sectional ‘healthy’ discovery population cohorts with age as a phenotype to construct a predictive model for an organism’s age and subsequently validate it in a separate cohort. Integrating these BA predictors with digital representations of individuals, often termed ‘human digital twins,’ refers to creating a dynamic computational model that captures an individual’s (near) real-time clinical, molecular, and lifestyle data. This model is continuously updated as new data become available, enabling healthcare providers and researchers to simulate potential interventions, predict disease risk, and personalise treatment strategies.

Human digital twins can incorporate data from wearable devices (e.g. heart rate, physical activity, and sleep trackers), electronic health records and multi-omics analyses (e.g. genomics, proteomics and epigenetics). By bringing these data streams together, digital twins allow for continuous modelling, simulation and prediction of health outcomes under various interventions or environmental exposures^2^. For instance, a digital twin can project how an individual’s BA will respond to a specific change in diet or exercise, thereby guiding preventive or therapeutic strategies in a timely manner.

However, implementing human digital twins in real-world healthcare settings poses several technical challenges. These include the need for secure data integration across disparate platforms, the development of scalable computational frameworks capable of handling high-volume longitudinal data, and the establishment of standards for data interoperability and privacy. Additionally, machine learning and systems biology approaches are required to dynamically update and validate the models as individuals’ data profiles evolve over time.

A critical element that enhances the precision of human digital twins is the incorporation of BA predictors. These predictors can act as **quantitative anchors** for the **digital twin’s ‘virtual health state,’** *allowing clinicians to track deviations from expected trajectories more accurately*. By regularly updating these predictors, clinicians and researchers can detect early signs of accelerated or decelerated ageing and refine intervention strategies accordingly, improving the predictive power of the twin. This synergy between digital twin technology and validated and robust BA measures stands to accelerate preventive medicine and decision-making, ultimately improving health span and quality of life^3^.

Based on the type of molecular data employed, these BA predictors can be classified into six categories: Telomere length (TL), epigenetic clocks, transcriptomic predictors, proteomic predictors, metabolomics-based predictors, and composite biomarker predictors. Among these, epigenetic clocks are considered to be the most accurate in predicting BA and other health phenotypes^4^.

Telomeres are repetitive DNA sequences capping chromosomes that shorten every time a cell divides; thus, TL is a conventional marker of biological ageing across various biological domains^5^. TL has been associated with BA, wellness and mortality risk of an individual^4–6^. Furthermore, TL has been proposed for some specific types of cancer^6^ and cardiovascular mortality predictions^7,8^. However, TL is hard to measure in clinical practice, and recent reports comparing TL with epigenetic clocks on non-symptomatic (or healthy) individuals found TL to be less informative^4,9–11^.

Epigenetic clocks are molecular tools based on 5mC methylation changes to a person’s DNA over time. These modifications, which can be influenced by various factors, including environmental exposures and lifestyle choices, can change over time and, therefore, be used to predict a person’s age. The ‘epigenetic clock’ premise is to link developmental and maintenance processes to biological ageing, giving rise to a unified theory of the life course of an organism^12^. Epigenetic clocks are used in ageing research to identify potential interventions that could delay or reverse age-related changes and to understand the biological processes underlying ageing. They are also employed to study the relationship between^2^ epigenetic changes and various age-related diseases and conditions, such as cancer and cardiovascular disease. Epigenetic clocks are not yet widely used in clinical practice but show promise as a way to measure biological ageing and identify interventions that may be able to improve health outcomes. There is a rapidly growing number of epigenetic clock estimators built for distinct purposes^13,14^, and their application potential, together with the other omics clocks, has also been discussed^15,16^. Proteomics, metabolomics, and multiple clinical biomarker readouts are used to predict BA, specific disease risks or phenotypes^4,17^.

Many of those predictors of age and fitness have already been proposed to be used in healthcare practice, and several companies have started to offer direct-to-consumer products in this context^18^. Their product implementation in clinical practice requires rigorous validation and simple and clear recommendations for both the healthcare practitioner and the individual. Hence, despite the large consensus of their premise, there are significant challenges to overcome to transfer scientific health and wellness tools into clinical practice^19^.

In this study, we aim to demonstrate the value of epigenetic clocks, proteomics, metabolomics, and multi-biomarker predictors in a unique longitudinal pilot cohort^20,21^ where all required data types (clinical parameters, epigenomics, proteomics, metabolomics, along with deep phenotyping and metadata) are available across multiple time points in a 13-month period. Aligned with the Physiome^22^ and EDITH^23^ projects we recognise the significance of the multiscale modelling hierarchy that forms system physiology and whole-body functions. Therefore, in alignment with multi-scale models of physiology, we also aim to explore how incorporating multi-omics-based ageing clocks into human digital twins can offer a hierarchical understanding of an individual’s state—from cellular and molecular processes to organ-level function and overall clinical risk. Our objective is *to assess the feasibility of operationalising the concept of biological ageing in conjunction with human digital twins*, ultimately enhancing our ability to provide tailored and adaptive insights into individual health trajectories.

## Results

### Study design and clocks overview

**Fig. 1:**
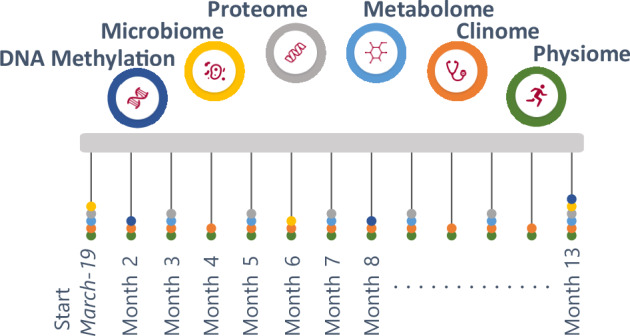
Study design of the IAM Frontier. Longitudinal comprehensive data were collected over the course of the study including monthly physiological and clinical biochemistry data (13 time points), bimonthly proteomics and metabolomics data (7 time points), and 6-monthly DNA methylation and microbiome data (3 time points). The study participants consist of 15 males and 15 females, with a (chronological) age range of 45–59 years old.

From March 2019 to March 2020, the IAM Frontier study collected monthly samples and data (via online questionnaires and wearable sensors) from 30 healthy (no diagnosed chronic diseases, no self-reported illnesses except hypertension) and highly motivated individuals. An intake interview was performed to check whether the volunteers fit the inclusion and exclusion criteria (health, age, sex balance) and to assess their motivation to join and stay within the study, as the study required monthly site visits for sample collection, continuous wearing of sensors and weekly questionnaires. All individuals were followed up with study doctor visits to inform them about their health status (from the clinical grade biomarkers), as well as provided with genetic counselling with certified personnel when necessary. The study design is illustrated in Fig. 1.

In this study, we first explore the global pattern of the values resulting from a multitude of ageing clocks and examine their utility in predicting personal wellness, health, and BA. These clocks were computed for all the IAM Frontier individuals, utilising their clinical biochemistry and physiological data (such as blood pressure, weight and height), DNA methylation, metabolomics, and proteomics measurements. Further, we investigate how these clocks predict outcomes across different time points, leveraging the longitudinal nature of the IAM Frontier study. Individuals with interesting findings are subjected to further investigation to demonstrate the importance and the relevance of the omics clocks as personal health assistants, capable of monitoring and assisting the clinical examination and diagnosis.

**Fig. 2:**
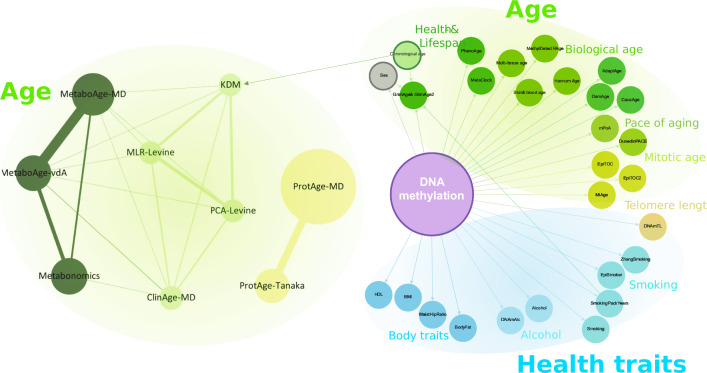
The overview of published clinical and omics ageing clocks and predictors discussed in this article. The predictors are grouped based on their purpose on predicting age, health traits, and telomere length. Each node represents one predictor connected to another. In the left-hand panel, the edges represent the ratio of shared parameters, e.g. there are 26 shared parameters between the MetaboAge-vdA clock and the MetaboAge-MD clock, hence the thickest edge. The size of each node represents the total number of parameters used in each clock. The right-hand panel shows all DNA methylation predictors discussed in this study. Edges indicate the parameters used for each predictor. Due to the high number of DNA methylation sites used by the predictors, nodes and edges are shown in standard sizes, with no relation to the number of features used or shared.

Figure 2 shows the overview of ageing clocks that can be estimated from various omics measurements. We categorise these clocks according to their utility in predicting (1) BA, (2) blood counts, (3) health traits such as smoking status, alcohol consumption and body mass index (BMI) and (4) plasma protein levels. By using DNA methylation data, 13 clocks aim to predict age, one clock to predict blood counts, 11 clocks to predict health traits, and seven clocks to predict plasma protein levels. In addition, three clinical biochemistry clocks, three metabolomics, and two proteomics clocks can also be used for predicting BA from clinical biochemistry data, NMR metabolomics, and Olink proteomics measurements. In this study, we focus on investigating the ageing clocks that are particularly useful in predicting BA.

In general, in each data type, different ageing clocks use a different set of features. The features used in ageing clocks will further be referred to as variables. Figure 2 shows that there are some variables that are shared and utilised in more than one clock, as demonstrated by the edges between clocks (see multiple linear regression (MLR)-Levine and Klemera and Doubal method (KDM)). It is also possible that these variables are shared across different data types, for example between the MLR-Levine and the Metabo-MD, where albumin is utilised in both clocks. Albumin and several other variables are present in the clinical biochemistry and metabolomics data; they refer to the same clinical compounds but are measured by different technologies. Since our study focuses on ageing clocks that aim to predict BA, it is also worth noting that there are several omics ageing clocks that include CA in their algorithm, for example, GrimAge and KDM. GrimAge also includes sex and plasma protein levels predicted from the DNA methylation data in its calculation.

Epigenomic, metabolomic and proteomic data are high dimensional. The IAM Frontier study collected the DNA methylation measurements of more than 850,000 sites per sample using Illumina’s Infinium MethylationEPIC BeadChips as well as 1068 proteins and 249 metabolites were measured in the metabolomics and proteomics data of IAM Frontier, respectively. Large numbers of variables are used in ageing clocks working with these data types. Although several epigenetic age clocks based on only a few loci have been proposed^24,25^, they tend to be less accurate. The most widely used epigenetic clocks and predictors incorporate between 71^26^ and 1030 CpGs^27^. In comparison, ageing clocks based on clinical biochemistry data or other omics data (metabolomics, proteomics) require nine to 203 parameters^28–31^.

### Global analysis of omics clocks and predictors

We implemented 26 BA clocks in this study, see Table 3 in ‘Methods’. Some of the clocks were not further investigated due to showing unrealistic predictions, unavailable model coefficients (e.g. the model intercept coefficients are not published in the MetaboAge-MD clock), or unavailable variables (e.g. due to differences in the omics technology, around 80% of the required metabolites used in the Metabonomics clock were not measured in the IAM Frontier dataset)^25,29,32–38^. Different sets of variables with different sizes are involved in each clock. The included algorithms do not constitute an exhaustive list but were selected in their applicability to the IAM Frontier study with high confidence due to data availability and matching underlying assumptions.

**Fig. 3:**
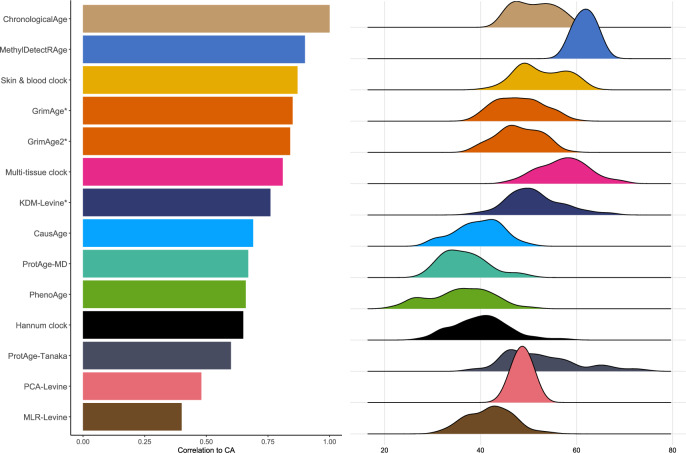
Density plots and correlations of BA predictions. MethylDetectRAge and Skin & blood clock have the highest correlations with the chronological age (CA) (left panel); the Skin & blood clock predicted age gives the most similar kernel density as CA, both in position and shape (right panel).

Although the study’s main objective is focused on BA prediction, CA is still incorporated in some analyses. While CA may not provide the most robust description of human ageing, it offers the most convenient way to calculate age and is perceived as a standard measure of ageing. Stratification by CA group is also often done, for example in clinical reference intervals^39,40^. For this reason, we believe it is valid to compare the predictions of BA with the subjects’ CA; see the Pearson correlation coefficients in Table 1. The majority of the clocks shown in Table 1 are significantly correlated with CA; MethylDetectR and the Skin & Blood clock give the highest correlation coefficients, $R$= 0.90 and $R$= 0.87, respectively. This is not unexpected, as both clocks were developed to predict CA. The kernel densities of the predicted BA as well as CA are shown in Fig. 3. The predicted Skin & Blood clock age gives a similar kernel density as the CA, both in the position as well as the shape. From this perspective, we may suggest this clock as the best tool to measure individuals’ chronological ageing. However, the correlation between CA and predicted age among all measured biological clocks is the highest for MethylDetectRAge, another CA predictor (Pearson’s $R$= 0.91), although its predictions appear to be slightly shifted towards older ages in our dataset. GrimAge, with $R$= 0.85, gives a position of the kernel similar to the CA, but with a different shape. GrimAge aims to predict lifespan and healthspan, and as a remark, it also incorporates CA in its calculation, unlike the other clocks, which are solely based on DNA methylation markers, other omics, or clinical measurements. The same remark also applies to the KDM-Levine clock, with $R$= 0.76 and an identical position of the kernel density as compared to the CA.

**Table 1:** Correlation between predicted age and chronological age

Predictor	Corr. coefficent	*p*-value
MethylDetectRAge	0.90	2.0E-30*
Skin & blood clock	0.87	3.5E-26*
GrimAge	0.85	1.1E-23*
GrimAge2	0.84	2.7E-22*
Multi-tissue clock	0.81	1.6E-19*
KDM	0.76	2.2E-16*
ProtAge-MD	0.67	2.2E-16*
PhenoAge	0.66	3.8E-11*
Hannum clock	0.65	4.8E-11*
ProtAge-Tanaka	0.60	2.2E-16*
MetaClock	0.56	8.7E-08*
PCA-Levine	0.48	2.2E-16*
MLR-Levine	0.40	1.4E-15*
ClinAge-MD	0.37	1.2E-13*
EpiTOC	0.25	2.4E-02*
MiAge	0.20	7.6E-02
MetaboAge-vdA	0.18	8.6E-03*
mPoA	0.18	1.1E-01
DunedinPACE	0.15	1.7E-01
MetaboAge-MD	0.08	2.6E-01
EpiTOC2	0.04	7.4E-01
CausAge	0.69	1.8E-12*
DamAge	0.62	1.3E-09*
AdaptAge	0.49	3.6E-06*

*Significant correlations are displayed with an asterisk (at *p* < 0.05). GrimAge and KDM incorporate chronological age in their calculation.

We performed different data exploration approaches to compare the BA predictions of each individual who participated in the IAM Frontier study. At each BA predictor, we observe smaller within-person predictions at different time points (focusing on *inter-individual variability*) than the between-person predictions at the same time point (focusing on *intra-individual variability*). The small inter-individual variabilities, which imply to large intra-class correlation (ICC) values, reflected by high ICC values, indicate that predictions for the same individual are more consistent over time than those observed between peers. This pattern aligns with the characteristics of standard clinical biomarkers measured longitudinally.

**Fig. 4:**
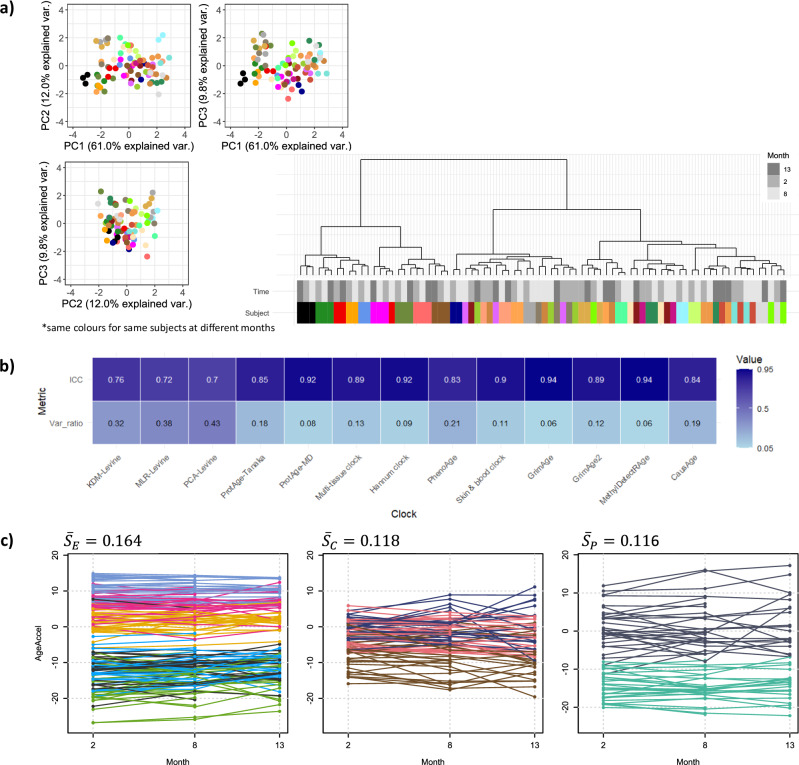
Longitudinal exploration of BA predictions in three different omics modalities. **a** Unsupervised analysis of BA predictions (PCA and cluster analysis) for all subjects in three shared time points, **b** ICC and *Var_ratio* of each BA clocks; large ICC and small *Var_ratio* indicate high within-person similarity, **c** Age acceleration of all IAM Frontier participants in different clocks; epigenetics clocks with ${\bar{S}}_{E}=0.164$: multi-tissue clock (pink), skin & blood clock (yellow), henoAge (green), GrimAge2 (orange), MethylDetectR (steel blue, top), CausAge(light blue, bottom) and Hannum clock (black). Clinical clocks with ${\bar{S}}_{C}=0.118$: MLR-Levine clock (brown), PCA-Levine clock (light pink), and KDM clock (blue). Proteomics clocks with ${\bar{S}}_{P}=0.116$: ProtAge-Tanaka (grey) and ProtAge-MD (tosca).

Our results show ICC values ranging from 0.70 to 0.94, while the ratios of intra- to inter-individual variability (*Var_ratio*) span from 0.06 to 0.43, as illustrated in Fig. 4b. In contrast to ICC, a higher *Var_ratio* suggests greater divergence in within-person predictions. Consistent patterns were also observed in the unsupervised analyses (Fig. 4a), where BA predictions from the same individual consistently clustered together, reinforcing the notion that within-person predictions are more similar than those between individuals. When we examine individual physical condition, clocks with lower ICC, such as PCA-Levine and ProtAge-Tanaka appear more sensitive to fluctuations in self-reported health status, as measured through weekly questionnaires on physical complaints. This is further supported by the pairwise correlation analysis between health trends and the average BA predictions from each clock (Supplementary Fig. 5b). While these clocks show less within-person stability, they may be better suited for capturing short-term biological changes associated with acute physiological changes. These findings support an analytical approach that emphasises inter-individual differences while also exploring the variability within individuals across time. For instance, Fig. 4c presents age acceleration—defined as the difference between predicted BA and CA—at time points two, eight, and 13, which are common across all omics and clinical data. Distinct predictions were evident across different clocks, with observable fluctuations across time points.

Within the same clock, we also observe that the between-individual fluctuations are larger than the within-individual’. We calculated the stability index (${\bar{S}}_{k},$ for each clock category $k$) based on these fluctuations,

1 $${\bar{S}}_{k}=\frac{1}{N}\mathop{\sum }\limits_{i=1}^{N}{\widetilde{S}}_{i},{\rm{with}}{\widetilde{S}}_{i}=\frac{1}{{mean}({d}_{12}^{i},\,{d}_{13}^{i},\,\ldots ,\,{d}_{n-1,n}^{i})}$$

and ${d}_{12}^{i},\,{d}_{13}^{i},\,\ldots ,\,{d}_{n-1,{n}}^{i}$ are the pairwise differences of BA from different time points at each subject $i$. For easier interpretations, we normalised the score to 0–1 range with a larger index indicating less fluctuations and hence, more stable predictions. The results reveal that epigenetic clocks provide the most stable predictions, followed by clinical data and proteomics. This suggests that the rate of change in each layer affects the reliability of BA predictions over time. Therefore, DNA methylation data should be used in conjunction with more recent clinical and proteomics data to produce stable BA predictions. The individual stability index, calculated for each clock category, further supports the finding that epigenetic clocks consistently yield more stable within-individual predictions (see Supplementary Fig. 6). Clinical and proteomic measures appear more sensitive to short-term influences such as lifestyle, medication, and other external factors. The age acceleration predictions of GrimAge, the Skin & Blood clock, KDM-Levine, PCA-Levine, and ProtAge-Tanaka are close to zero, i.e. their predicted BA values are close to the corresponding CAs. Referring to the estimated kernel density in Fig. 3, the predicted values of these clocks are similar to the CA with reference to the location.

### Individual analysis of omics clocks and predictors

**Fig. 5:**
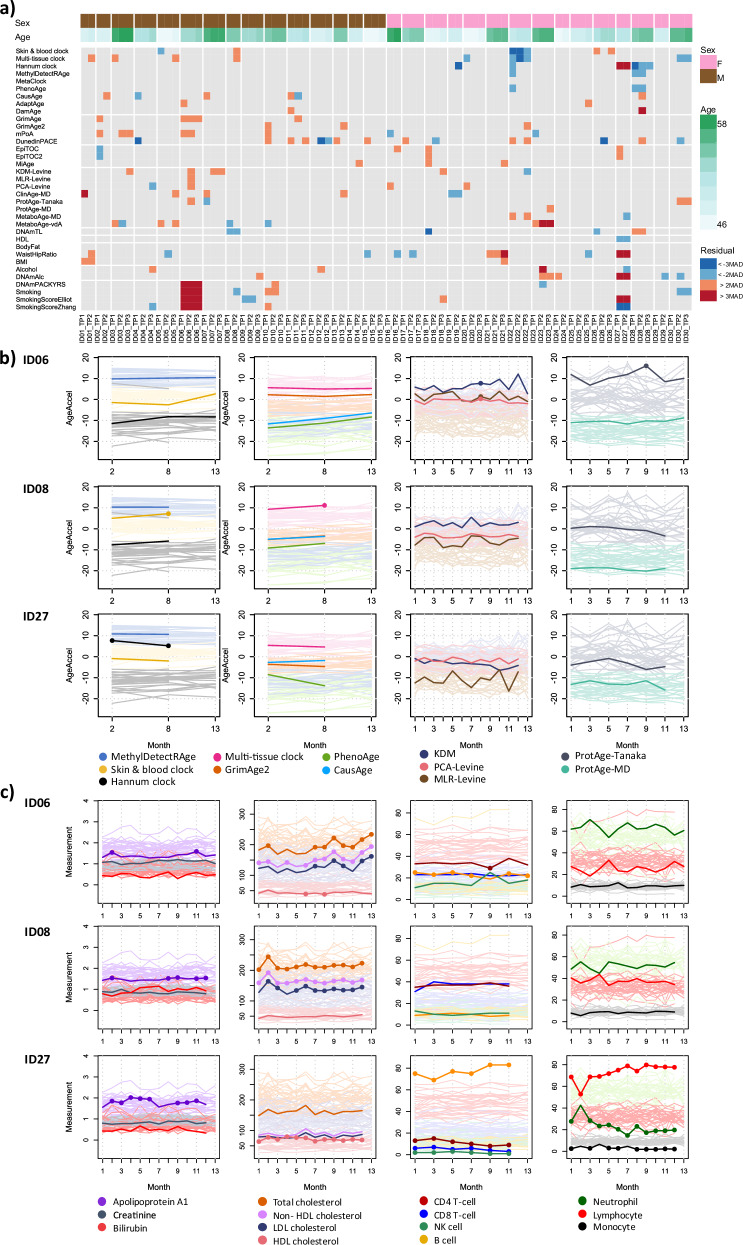
Individual analysis of BA predictions in all available time points. **a** Reduced ordered heatmap based on MAD thresholds. **b** Age acceleration of ID06, ID08, and ID27. Prediction values that are significantly different from the rest of the cohort are marked with full circles. **c** Clinical and blood cell profiles of ID06, ID08, and ID27. Measurements that are outside the cohort reference intervals are marked with full circles. The blood cell counts are shown in percentages.

We continue the analyses by investigating the BA and health traits predictions in all IAM Frontier participants. Due to the small sample nature of the IAM Frontier study, we are able to examine the individual predictions resulting from all clocks. Figure 5a shows the ordered heatmaps of the predicted BAs as well as the health trait predictions in all individuals at time points two, eight, and 13. These were selected because the results of all clocks are available at these time points. The MAD thresholds were computed and were used to give the colour annotations. Therefore, only deviating predictions appear coloured in the figure. We can observe distinct patterns for ID06, ID08 and ID27, detailed below. In Fig. 5c, we show the clinical laboratory profiles of these individuals.

ID06 shows very high smoking predictions. ID06 is a current smoker, indeed, and hence has a higher risk of developing cardiovascular diseases (CVD) and diabetes (Supplementary Fig. 3 of CVD and diabetes disease risk scores). From the individual plots in Fig. 5b, we also observe that the BA predictions of ID06 increase over time for some of the ageing clocks, such as in the Skin & Blood clock and PhenoAge. This individual was also biologically older at all time points according to the KDM and ProtAge-Tanaka clocks. However, the clinical laboratory profiles of ID06 show an increasing trend of the lipid measurements, in line with this individual’s increased risk of CVD and diabetes. Interestingly, ID06’s B-cell percentages are higher than the normal range, and the CD4 T-cell and lymphocyte counts are low. This could show that this individual might also have problems with antibodies and the immune system. In the questionnaires, ID06 did not report major health burdens except that this individual felt having low energy, tense muscles, and constant fatigue, with the latter two commonly reported among the study participants.

ID08 appears as the only individual with predicted shortened telomeres across both measured time points based on the DNAmTL clock, as seen in Fig. 5b. This result may indicate premature ageing. In addition, in almost all the applied epigenetic clocks, this individual was also predicted to be epigenetically older at the second time point compared to the first (see Fig. 5b). The lipid (cholesterol) profiles of ID08 (see Fig. 5c) show an alarming status; the measurements are out-of-normal range at almost all time points. Other clinical parameters are still within the normal limits, although the percentages of CD8 T-cell and lymphocyte are high compared to the peers. Based on the weekly questionnaires, during the course of this study, this individual experienced constant fatigue, diarrhoea, numbness, indigestion, sleeping problems, stuffy nose, and blurry vision, see Supplementary Fig. 5a. Although ID08 does not appear to report all of these health burdens at a significantly higher level than the other study participants, together with the rapid epigenetic ageing and high lipid profiles observed, they might provide additional support to the premature ageing condition predicted by the DNAmTL clock.

ID27 appears to be different in our analyses in many ways, although most of the epigenetic, proteomic and clinical ageing clocks predict this individual to be in a healthy state—healthier and biologically younger than the peers. However, ID27 shows very strong outlier BA predictions corresponding to the Hannum clock as well as very different smoking and health trait predictions. It is the only subject with a completely distinct Hannum age acceleration compared to the peers at all time points, epigenetically younger at the second time point as compared to the first across all clocks, and with conflicting smoking predictions (this individual was not a current smoker). The BA predictions of the clinical and proteomics clocks also show a general decreasing trend over time, where ID27 was biologically younger than chronologically, according to some of those clocks. In addition, from the DNA methylation and proteomics analyses, this subject is discovered to be far outside the main cluster in the corresponding PCA plots (Supplementary Fig. 4a, b). When looking at DNA methylation predicted blood cell composition, we observe a strongly increased proportion of B cell (Supplementary Fig. 7). The finding is confirmed by actual blood counts showing the same abnormalities, clearly indicating a haematological problem. This explains why ID27’s age predictions appear as extreme outliers according to Hannum’s clock, as it is sensitive to blood composition changes, and may also explain the conflicting and unexpected outlier predictions obtained for several health traits such as smoking and alcohol intake, among others. Throughout the study period, ID27 reported constant medium backpain, a slight headache and blurry vision.

## Discussion

Age stands as the principal risk determinant for ailments, impairments, and diseases. The pursuit of mitigating age-related illnesses and extending the healthful years of life has led to the innovative idea of directly targeting the ageing process to restore physiological functionality. Achieving this ambitious goal requires the precise assessment of BA and the pace of ageing at the molecular level. Building on the latest breakthroughs in high-throughput omics technologies, a novel suite of tools has emerged for the quantitative analysis of biological ageing. By leveraging data from various domains—such as epigenomics, proteomics, and metabolomics—and employing machine learning techniques, ‘biological ageing clocks’ have been constructed. These clocks have proven their ability to pinpoint potential biomarkers of biological ageing, offering unprecedented insights into the ageing process and laying the groundwork for anchoring digital twin models to health trajectories.

A recent review paints today’s landscape of BA prediction algorithms using omics technologies^41^. These methods are claimed to better represent the biological state of an organism than the CA. Ageing clocks for BA are also starting to appear as commercial products, serving as a window into the personal health status. In this manuscript, we use a unique deep-phenotyping dataset that allows us to perform and analyse multiple BA predictors using different data types side by side. The longitudinal nature of the data enables us to study the individuals’ fluctuations between time points, exploring their changes in BA predictions over time. We optimise our analyses using the extensive set of technical replicates generated to estimate technical variation in our dataset and design our analyses correspondingly (see ‘Methods’ and Supplementary Material Section 1).

The BA analyses in the IAM Frontier data shows that **BA predictions can fluctuate over time**; both an increase and a decrease might happen depending on the biological conditions **rather than monotonically increase by time, as in CA**. Repeated measurements over time are highly valuable, as outliers can also be caused by normal biological fluctuations. The stability index, which quantifies the consistency of BA predictions across different time points, also indicates that **ageing likely manifests at different rates across various omics layers**. As represented within the multi-dimensional modelling of digital twins, ageing is a multi-dimensional—or multilayer—process. In the physiome hierarchical framework, organs are mapped from cells to functional tissue units (FTUs) and then to the organ level (a similar approach is taken in EDITH and other digital twin frameworks)^22,42^. Extending this multi-level structure into **BA** enables real-time simulation of age-related changes across various biological scales. In the context of ageing, *BA predictions can serve as quantifiable indicators for analysing deviations in ageing at the cellular or even FTU levels, thereby refining the digital twin’s ability to capture early signs of physiological decline*. Understanding stability is crucial for determining the appropriate periodicity of data collection in each omics layer to ensure accurate, multi-scale integration into digital twin models.

In our individual analysis, most BA predictions were ‘**stable**’, that is, within the expected range of variability across the available time points. However, there were individuals whose predictions do change more than expected over time, and where this is the case, they are often consistent across multiple BA predictors and time points. A comprehensive exploration of the BA prediction differences between individuals of our study leads to **three individuals with distinct BA predictions** as compared to their peers.

The BA predictions of *ID06*, *ID08*, and *ID27* provide a compelling illustration of how multi-omics data can not only support standard laboratory findings but also pinpoint subtle physiological deviations well before overt clinical symptoms emerge. In a digital twin context, these detailed omics layers and BA predictors could be integrated into a computational model that flags abnormal trajectories in real time, prompting earlier diagnostic follow-up. For example, ***ID08*’s consistently short telomeres and abnormal lipid profiles could trigger predictive simulations about cardiovascular risks**, while ***ID27*’s epigenetic deviations could inform more targeted immunological assessments**. These instances highlight the promise of digital twin models to capture and interpret diverse biomarker changes as they unfold.

These strong alterations may be what impacted some of the reported epigenetic health trait predictions. Of note, according to the developers of those health trait predictors, they should currently only be used at a population level and are not yet supposed to provide reliable predictions at the individual level. Training data from larger-scale cohorts, *including diseased individuals*, will be required to refine these additional health trait predictors and enable their clinical use^43^. Irrespectively, the clearly abnormal epigenetic measurements and predictions of *ID27* would undoubtedly also have led to further clinical tests if detected in a wellness- or preventative healthcare setting, revealing the ongoing but previously undetected pathological process.

Our findings demonstrate the value and potential of epigenetic predictors and BA estimators, particularly for risk assessment and early detection. Several newer versions of these epigenetic predictors, introduced and integrated in this study, are at least on par with their earlier models, while still offering valuable new benefits. Our integrative analysis with self-reported health complaints further shows important distinctions between clock types. Clinical and proteomics-based clocks, while exhibited slightly lower overall stability (with the lowest ICC of 0.76), more accurately captured short-term fluctuations in health status and physical complaints compared to epigenetic clocks. A greater number of health complaints showed significant associations with changes in these clocks’ BA predictions, suggesting they may be particularly sensitive to acute, short-term physiological states. In contrast, epigenetic clocks appeared considerably more stable within individuals but were less reflective of week-to-week physical variations, possibly reflecting more stable, long-term biological aging processes.

Taken together, these BA clocks can finally reveal unexpected deviations and inform about the probability of having or developing a disease, for example shown here by individual *ID27*. Moreover, they can serve as the cornerstone in anchoring the implementation of human digital twins that model and simulate biological processes in real-time.

Recent advancements demonstrate the growing potential of digital twins in capturing the complex dynamics of human ageing^44^. Incorporating BA clocks into digital twins demands the creation of ‘**biological-age-sensitive**’ modelling paradigms in which normal age-related changes are distinguished from genuine disease progression. As illustrated by our cohort findings, clocks derived from epigenetics, proteomics, or metabolomics can parameterise tissue- and organ-level simulations, improving model fidelity. By regularly updating these BA-based parameters, digital twins can track the natural ageing continuum while highlighting deviations that may signal the onset of pathology. Hence, considering that BA clocks have the potential to reflect the age/health status of the tissue or organ from the perspective of the modality (methylation, proteomics), they have the potential to parameterise the tissue or organ level models better.

In the future, when epigenetics and multi-omics data of patients are routinely captured, researchers and clinicians could integrate these data into the digital twin’s analytics pipeline, calibrating BA models using validated clinical outcomes, and finally performing iterative updates as new biomarker measurements become available. This would us to optimise personalised medical treatments, and refine preventive healthcare strategies. However, several challenges remain before the seamless deployment of biological-age-sensitive human digital twins in clinical practice, including (i) data standardisation and interoperability across disparate omics platforms, (ii) ensuring model accuracy and generalisability given high-dimensional datasets, (iii) managing the computational burden of real-time or near-real-time updates, and (iv) establishing robust data privacy, security, and regulatory frameworks. Addressing these technical and ethical hurdles will be essential for enabling BA-driven digital twins to evolve from conceptual prototypes into mainstream clinical tools.

Currently, the extensive and complex preprocessing procedures required to obtain high quality results from omics data are still hindering many of the corresponding predictors from developing their full clinical potential, particularly at the individual level. However, with the field growing and maturing, we argue that BA predictions should be considered as crucial biomarkers that can anchor digital twin models and well-complement routine medical tests and CA.

Omics data have already begun to enter clinical practice^45^, making omics-based BA predictions feasible. However, despite their high-dimensionality, omics datasets often suffer from limited sample sizes. In this IAM Frontier study, for instance, the small sample size (*n* = 30) presents challenges for generalising the results and reduces statistical power. To enhance the reliability of the analyses, several measures were implemented, including the use of the median absolute deviation (MAD) to identify and mitigate outliers. This approach is more robust and less influenced by extreme values. Additionally, downstream analyses were not solely reliant on aggregate results, which can be skewed by outliers, but also incorporated subject-level analyses. This ensures that potential biological findings are not overlooked or diluted, as can happen in population-level analyses. By adopting this dual approach, we mitigate some of the risks associated with small sample sizes and improve the robustness of the conclusions.

Once omics technologies have been fully incorporated into the medical routine, BA predictions will likely become standard measurements regularly discussed between patients and medical professionals. A novel personalised value to identify the ‘normal’ BA for an individual could also be estimated, together with common clinical measures, to provide precise individual interpretations^46,47^. In line with the clinical trial frameworks, epigenetic age measurements are also being implemented more frequently in studies investigating interventions for age-related diseases^48^. In oncology practices, consistent findings have been observed that individuals with accelerated epigenetic ageing often display a higher risk of developing various cancers, suggesting that regular BA monitoring may help identify high-risk patients for targeted screening^49,50^. In industry, this momentum is also already apparent for startups and biotech companies that offer epigenetic-based BA testing panels (https://gero.ai/, https://www.elysiumhealth.com/, https://glycanage.com/), demonstrating commercial readiness and patient demand for these emerging biomarkers. Despite this promise, the field remains in its early stages. Nevertheless, through continued large-scale validation, alignment with healthcare policies, and education of both clinicians and patients—BA predictions are poised to become essential tools for preventive and precision-oriented medical care.

Further multi-scale longitudinal studies—including broader and more diverse populations, as well as external factors such as environment, lifestyle and real-time physiological metrics—will undoubtedly strengthen the validation of BA predictions at the personal level. In a digital twin context, such datasets would refine the capacity to model complex interactions and generate highly individualised forecasts. By integrating epigenomic, proteomic, and metabolomic data into machine-learning-enhanced simulations, future digital twins could differentiate natural ageing processes from disease-driven changes with greater precision. Ultimately, these innovations hold the potential to accelerate risk stratification, guide interventions, and transform how clinicians and patients collaborate on preventive and precision-oriented healthcare. Such studies would allow for simulations in complex scenarios where multiple variables interact, providing insights into the intricate dynamics that influence health and ageing. As the field of biological ageing advances, the construction of BA using ageing clocks employing diverse data sources such as epigenomics, proteomics, and metabolomics could be proven effective in uncovering novel biomarkers of biological ageing. Future endeavours to weave multi-omics into ageing clocks are poised to not only broaden our grasp of the molecular signatures that characterise ageing but also enhance the predictive powers of these models. The involvement of machine learning in harnessing diverse datasets—both molecular and environmental—would also advance the models and more accurately reflect how individuals respond to interventions or health risks. Moreover, as demonstrated by this study, where individual lifestyle factors were shown to influence ageing, incorporating these and potentially other external factors will be crucial. The continued expansion of this integrative approach is expected to yield more precise and actionable insights, solidifying the role of ageing clocks as indispensable tools. Ultimately, this will lead to robust multi-scale modelling of human digital twins, further advancing the evolving landscape of personalised medicine and ageing research.

In summary, the convergence of BA clocks with human digital twin methodologies offers a powerful new paradigm for personalised healthcare. Through the dynamic integration of molecular and clinical data, digital twins can highlight early biomarkers of ageing and disease, optimise treatment strategies, and ultimately extend healthspan. Our findings underscore the feasibility of this approach and pave the way for future research into scalable, interoperable, and privacy-conscious platforms that will bring BA-driven digital twin models closer to routine clinical practice.

## Methods

### Study design and participants

The IAM Frontier study is a unique longitudinal cohort study that ran for 13 months in 30 healthy individuals, consisting of 15 male and 15 female participants^21^. The 13-month study duration was chosen to cover the seasonal fluctuations that might occur over a 1-year period. The study specifically targeted the employees of the research organisation VITO within the age range of 45–59. A major reason for the selection of this group of employees was that as they are part of a research organisation, they are expected to be more open to research-grade technologies and interventions. The age range was selected because the highest prevalence of onset of chronic diseases occurs from the age of 45–65^51^. Individuals were selected based on the following inclusion criteria: not suffering from a chronic disease, diagnosed and currently followed-up by a medical specialist: asthma, chronic bronchitis, chronic obstructive pulmonary disease, emphysema, myocardial infarction, coronary heart disease (angina pectoris), other serious heart diseases, stroke (cerebral haemorrhage, cerebral thrombosis), diabetes, cancer (malignant tumour, also including leukaemia and lymphoma). At monthly visits, a range of samples (whole blood, plasma, urine, stool) were collected and sent to accredited laboratories and comprehensive multi-omics and clinical biochemistry data were assessed. Self-administered questionnaires on, for example, health conditions and physical activity were also completed by the participants. In this article, we analysed data from the IAM Frontier study, which included DNA methylation data, clinical biochemistry, proteomics, metabolomics and physical examinations data.

### Sample collection

The sampling of the IAM Frontier study took place between March 2019 and March 2020 and included the collection of human biospecimen and digital data. During the 13 months of the study, the participants donated blood, urine and stool samples at monthly visits. These samples were collected after overnight fasting for at least 8 h. The urine and blood samples (in EDTA-, citrate-, and serum-vacutainers) were transported to the clinical laboratory at room temperature within 6 h after collection. Peripheral blood mononuclear cells (PBMC) were isolated from EDTA-blood samples, and PBMC pellets were stored at −80 °C till the DNA extraction. At monthly visits, clinical tests and health examinations such as blood pressure, body height, weight, and abdominal circumference measurements were performed by accredited labs and appointed doctors. At bi-monthly visits, plasma samples were taken, and omics (proteomics and metabolomics) measurements were assessed. At months 1, 6 and 13, the PBMC samples were used to measure DNA methylation. At month 13, only 20 participants were able to donate samples due to the start of the COVID-19 pandemic. Table 2 presents an overview of the sample collection. All samples have been collected in accordance with the applicable Belgian regulations regarding the use of human body material for scientific research (Belgian Law on use of human body material, 2008) and the Belgian Royal Decree on biobanks (*Het Koninklijk Besluit betreffende de biobanken. Belgisch Staatsblad 05.02.2018. Brussels (2018)*). All participants signed an informed consent and the study was approved by the ethical committee of the Antwerp University Hospital (RegN°: B300201938600). The research was conducted in compliance with the Declaration of Helsinki and all relevant ethical guidelines.

**Table 2:** Data collection of the IAM Frontier study

Data	Measurement technique	Timepoints – month	Type of sample	Total samples	Remarks
DNA methylation	Illumina Infinium MethylationEPIC BeadChips	2-8-13	PBMC	96	20 samples at month 13, 16 technical replicates
Clinical and physiological	Clinical biochemistry, blood cell counts, health examination	1-2-3-4-5-6-7-8-9-10-11-12-13	Whole blood, serum PB, fasting urine	380	20 samples at month 13
Metabolomics	NMR - MS	1-3-5-7-9-11-13	Plasma	200	
Proteomics	LC-MSMS -PEA	1-3-5-7-9-11-13	Plasma	200	
Microbiome	Illumina MiSeq	1-6-13	Stool	20	16 samples at month 13

### DNA methylation assay

The DNA methylation assay was carried out using Diagenode Epigenomic Services (Vienna, Austria, Cat No. G02090000). PBMC samples were sent for DNA methylation profiling using the Illumina Infinium MethylationEPIC array BeadChip (850 K) platform to analyse the methylation status of more than 850,000 CpGs per sample. This microarray covers ∼96% of CpG Islands and 99% of annotated RefSeq genes. We performed the DNA methylation data pre-processing and the corresponding details can be found in Supplementary Document Section 1.

### Analysis of epigenetic clocks and predictors

We applied 34 different epigenetic age and health trait predictors (see Fig. 2). The Skin & Blood Clock^52^, Multi-tissue Clock^53^, HannumAge^26^, DNAmTL^38^, PhenoAge^54^, GrimAge^27^, GrimAge DNAmPACKYRS and GrimAge protein levels (DNAmADM, B2M, CystatinC, GDF15, Leptin, PAI1, TIMP1) were obtained by Steve Horvath’s DNA Methylation Age Calculator available on http://dnamage.genetics.ucla.edu/. We also included the new version of GrimaAge, GrimAge2^55^. The MethylDetectR predictions (Age, Alcohol, BMI, HDL, BodyFat, Waist:Hip Ratio and Smoking)^43^ were calculated using the code available at https://zenodo.org/record/4646300. The methylation Pace of Age (mPoA)^25^ was estimated using the code available at https://github.com/danbelsky/DunedinPoAm38. The refined version of mPoA, DunedinPACE^56^ was also estimated. The MetaClock^34^ code was received by e-mail from the author Morgan E Levine. EpiTOC scores were calculated using the code available in the corresponding publication^35^. EpiTOC2^36^ scores were calculated using the code available from 10.5281/zenodo.2632938, and MiAge^37^ scores were calculated using the code available from http://www.columbia.edu/~sw2206/softwares.htm. Alcohol predictions^57^ were generated using the *dnamlci* R package available from https://github.com/yousefi138/dnamalci. Elliot’s smoking score^58^, Zhang’s smoking score^59^ and EpiSmokEr’s smoking status^60^ were obtained using the R package *EpiSmokEr* available at https://github.com/sailalithabollepalli/EpiSmokEr. Causality clocks^61^ including CausAge, DamAge, and AdaptAge were estimated using the *biolearn* Python library available from https://github.com/bio-learn/biolearn/.

GrimAge, HannumAge, MethylDetectRAge, mPoA, as well as the smoking, alcohol and health trait predictors, were originally developed for whole blood measurements but have been shown to work well with PBMCs in our study and others^62–65^. Table 3 shows the list of clocks implemented in this study, including clocks predicted from clinical, metabolomics, and proteomics data (see the next section).

**Table 3:** List of applied clinical and omics ageing clocks

Clock	Reference	Data type	Remarks
^‡^MLR-Levine^28^	Levine, ME. 2013. Gerontol A Biol Sci Med Sci.	Clinical	Predicts chronological/ biological age
^‡^PCA-Levine^28^			Predicts chronological/ biological age
^‡^KDM^31^	Klemera, P. and Doubal, S. 2006. Mech Ageing Dev.		
^‡^ProtAge-Tanaka^30^	Tanaka, T., et al. 2018. Aging Cell.	Proteomics	Predicts chronological/ biological age
^‡^ProtAge-MD^29^	Macdonald-Dunlop, E., et al. 2022. Aging.		Predicts chronological/ biological age
ClinAge-MD^29^	Macdonald-Dunlop, E., et al. 2022. Aging.	Clinical	Overestimated BA (median BA 750 years), not shown
MetaboAge-MD^29^	Macdonald-Dunlop, E., et al. 2022. Aging.	Metabolomics	Overestimated BA (median BA 600 years), not shown
MetaboAge-vdA^32^	van den Akker, EB., et al. 2020. Circ Genom Precis Med.		Underestimated BA (median BA –5000 years), not shown
Metabonomics^33^	Hertel, J., et al. 2016. Journal of Proteome Research.		Missing variables (only 22% vars. are available), not shown
^‡^Skin & blood clock^52^	Horvath, S., et al. 2018. Aging.	DNA methylation	Predicts chronological age
^‡^MethylDetectRAge^43^	Hillary, R. and Marioni, R. Wellcome Open Res.		Predicts chronological age
^‡^Multi-tissue clock^53^	Horvath, S. 2013. Genome Bioloy.		Predicts chronological/biological age
^‡^Hannum clock^26^	Hannum, G., et al. 2013. Molecular Cell.		Predicts chronological/biological age
mPoA^25^	Belsky, D., et al. 2020. eLife.		Predicts the pace of ageing
DunedinPACE^56^	Belsky, D., et al. 2022. eLife.		Predicts the pace of ageing
^‡^PhenoAge^54^	Levine, M., et al. 2018. Aging.		Predicts health- and lifespan
^‡^GrimAge^27^	Lu, A., et al. 2019. Aging.		Predicts health- and lifespan
^‡^GrimAge2^55^	Lu, A., et al. 2022. Aging.		Predicts health- and lifespan
Metaclock^34^	Liu, Z., et al. 2020. Aging Cell.		Predicts mortality
EpiTOC^35^	Yang, Z., et al. 2016. Genome Biology.		Predicts mitotic age
EpiTOC2^36^	Teschendorff, A.E. 2020. Genome Medicine		Predicts mitotic age
MiAge^37^	Youn, A., and Wang, S. 2018. Epigenetics.		Predicts mitotic age
DNAmTL^38^	Lu, A., et al. 2019. Aging.	DNA methylation	Predicts telomere length
^‡^CausAge^61^	Ying, K, et al. 2024. Nature Aging.		Predicts chronological/ biological age
DamAge^61^	Ying, K, et al. 2024. Nature Aging.		Predicts chronological/ biological age
AdaptAge^61^	Ying, K, et al. 2024. Nature Aging.		Predicts chronological/ biological age
GOLD BioAge^71^	Hao, et al. 2025. Adv. Sci.	Clinical	Predicts morbidity & mortality
GOLD ProtAge^71^		Metabolomics	Predicts morbidity & mortality
GOLD MetAge^71^		Proteomics	Predicts morbidity & mortality

^‡^Rows with double dagger symbol (‡) refer to clocks that are further investigated.

We also predicted blood counts using minfi^66^ and the Reinius reference dataset^67^, as well as IDOL using the Salas reference dataset^68^ with the code available from https://github.com/immunomethylomics/FlowSorted.Blood.EPIC.

The correlation between predicted and CA was calculated using Pearson’s correlation. We excluded technical replicates from the calculation of correlation coefficients.

For the longitudinal analyses of epigenetic predictions, we used the 16 technical replicates available from our study to estimate technical variation. Two technical replicates were generated within each of the three DNA methylation time points, and ten additional technical replicates of the two previous time points were generated at the third time point, providing detailed insight into technical variation influencing the predictions. We used the maximum absolute difference observed between technical replicates as a threshold to define potential biological differences across time points after also adding the chronological time that passed between time points. For example, the maximum absolute differences observed for the Skin & Blood Clock across technical replicates was 2.56 years (the reported median error in blood is 2.5 years^52^), and the threshold we used represents the maximum differences observed among technical replicates plus the chronological time that passed between the time points which are maximum 343 days (0.94 years).

For the global analyses of our dataset and the identification of biological outliers of interest based on our cohort, we introduced a mean absolute deviation (MAD) threshold of ±2*MAD and ±3*MAD across all biological replicates. In particular, ${\hat{y}}_{{ij}}\pm 2{MAD}$ and ${\hat{y}}_{{ij}}\pm 3{MAD}$, where ${\hat{y}}_{{ij}}$ is the individual BA prediction of subject $i$ at time point $j$. The MAD thresholds correspond to the 95th and 99th standard normal percentiles and are selected for their robustness in describing data with a relatively small time series as in the IAM Frontier study.

### Analysis of clinical, metabolomics, and proteomics clocks

The monthly clinical and bi-monthly metabolomics as well as proteomics data were used for predicting longitudinal BA using several published calculators. We applied the MLR model and principal component analysis (PCA) developed by Levine et al.^28^—they will be referred to as MLR-Levine and PCA-Levine—to the clinical data that consist of samples from 12 to 13 time points per individual. The model prediction involves both clinical and physiological measurements, including total cholesterol level, glycated haemoglobin, C-reactive protein, systolic blood pressure, forced expiratory volume (FEV) and cytomegalovirus (CMV). In the IAM Frontier data, FEV and CMV were not measured, but we imputed the values with the corresponding median from the original study^69^. The same clinical variables as in the PCA-Levine model were further used to predict the BA using the KDM^31^. Unlike the MLR-Levine and the PCA-Levine, KDM also incorporates CA in their estimation procedure. Similarly, we also computed other BA predictions based on similar clinical biochemistry variables developed by McDonald-Dunlop et al.^29^.

The proteomics and NMR metabolomics data consist of samples from six to seven time points per individual, where the samples were sent to the laboratory in four different batches. For the proteomics data, batch correction normalisation was done prior to the analysis to reduce the technical variation between batches/plates^70^. We performed PCA and multidimensional scaling analyses for the metabolomics data, where we did not observe any batch effects, so the raw metabolite abundances were used. In both datasets, there are twenty subjects with technical replicates spread across different time points. We performed a procedure for selecting the samples (between the originals and the replicates) by computing cosine similarity coefficients for all samples. The samples with the closest similarity to the rest of the individuals’ measurements were selected. Further, we predicted the BA using other published proteomics and metabolomics clocks: ProtAge-MD (from McDonald-Dunlop et al.^14^), MetaboAge-MD, ProtAge-Tanaka, MetaboAge-vdA, and Metabonomics^29,30,32,33^. Recently, the GOLD BioAge model was proposed as a Gompertz-based BA estimator using clinical and omics data^71^. We reference it here to highlight ongoing developments in this area.

### Software tools and programming language

The network (Fig. 2) was created in Gephi version 0.9.5^72^ and Cytoscape version 3.8.2^73^. All data analyses were conducted in the R statistical environment version 4.1.3.

## Supplementary information

Supplementary document_v2

## Data Availability

Due to participants’ privacy, the data is available upon request to the I AM Frontier Project Data Access Committee for further research (DataAccess.IAF@vito.be). The sensitive nature of the data does not allow it to be deposited into public repositories. We welcome collaboration with other research groups focused on personalised prevention, offering data sharing opportunities to advance research and improve healthcare outcomes.

## Code availability

The codes are available upon request from the corresponding authors.
