# Supplementary material for: From ageing clocks to human digital twins in personalising healthcare through biological age analysis
Source: NPJ Digit Med. 2025 Aug 21;8:537. doi: 10.1038/s41746-025-01911-9 (PMC12371080; doi:10.1038/s41746-025-01911-9)
Supplement: Supplementary file 1 — Supplementary document_v2 [file 41746_2025_1911_MOESM1_ESM.pdf]

Supplementary document

From Ageing Clocks to Human Digital Twins in Personalising Healthcare through Biological Age Analysis

1. DNA methylation data preprocessing

We performed extensive quality assessment of the DNA methylation data using Minfi's qcPlot and QCReport functions[1], plotCtrl of the ENmix package[2] and the QC metrics of Heiss & Just[3]. One out of the 96 samples did not pass the quality control checks and was removed from the dataset. The failed sample was a technical replicate. We also applied a sex check and performed SNP analyses to identify potential sample mixups.[3] No issues were detected in these analyses.

We filtered probes with a bead count of less than three in at least 5% of samples and probes with a detection p-value > 0.01 in at least one sample. Non-cg probes were also excluded. Probes mapping to sex chromosomes or SNPs were not removed as several epigenetic predictors make use of a subset of these probes. However, they were excluded for data visualization purposes during quality control and preprocessing and specifically accounted for where required, for example during batch effect correction (see below). The probe filtering reduced the number of used probes from 865,859 to 836,477.

We assessed the global distributions of the data using density plots (Supplementary Figure 1), the visualization of PCAs of methylation M-values and MDS plots of methylation Beta-values as well as unsupervised hierarchical clusterings using Euclidean distances and different linkage methods. The raw filtered dataset showed a light batch effect corresponding to the three timepoints of our study but were of good quality otherwise (see Supplementary Figure 1).

All samples from one particular participant of the study were identified as strong global outliers. These were samples from two timepoints and one technical replicate (n=3). The strongly altered DNA methylation profiles did not seem to be caused by technical issues but biological differences and were consistent over time. Therefore, these samples were not removed from the analyses. To avoid biasing the rest of the data because of the three strong outliers during preprocessing steps that borrow information from all samples, we performed our preprocessing pipeline (see below) twice: First, we removed all samples from the corresponding individual for preprocessing. Second, we repeated the same preprocessing procedure now also including the biological outlier samples. We then added the results of the outlier samples preprocessed in the second run back into the main dataset that was preprocessed without the outliers.

Technical replicates from all three timepoints (n=2 per timepoint) and also across timepoints (n=10) were used to assess performance, along with the data visualization procedures as described above. Density plots, PCA, MDS and hierarchical clusterings were repeated after every preprocessing step.

For background correction and dye-bias normalization we applied NOOB (normal-exponential convolution using out-of-band probes).[4] This method estimates the background mean intensity using out-of-band control probes which provide signals in the opposite fluorescent channel. NOOB effectively adjusts for differences in background distribution and average intensities between samples run on different arrays.[4] Subsequently, we applied BMIQ (Beta-Mixture Quantile normalization)[5] to adjust for probe type bias and perform intra-array normalization.

To assess batch effects, we performed singular value decomposition (SVD) of the DNA methylation M-values and Beta-values, excluding probes mapping to the sex chromosomes. The analysis revealed significant batch affects corresponding to array and slide. We employed the empirical Bayesian framework ComBat of the SVA package[6] to correct for the identified batch affects, using age as phenotype of interest and including sex as covariate as we needed to retain probes mapping to sex chromosomes in our dataset for downstream analyses using epigenetic predictors.

Data visualization as described above and unsupervised hierarchical clustering (see Supplementary Figure 2) confirmed that our preprocessing procedure successfully removed batch effects and samples of the same person always clustered together. Technical replicates also clustered very closely together. Biological subclusters of the same age and sex were visible, while no subclusters of any technical variables were present anymore after normalization and batch effect correction.

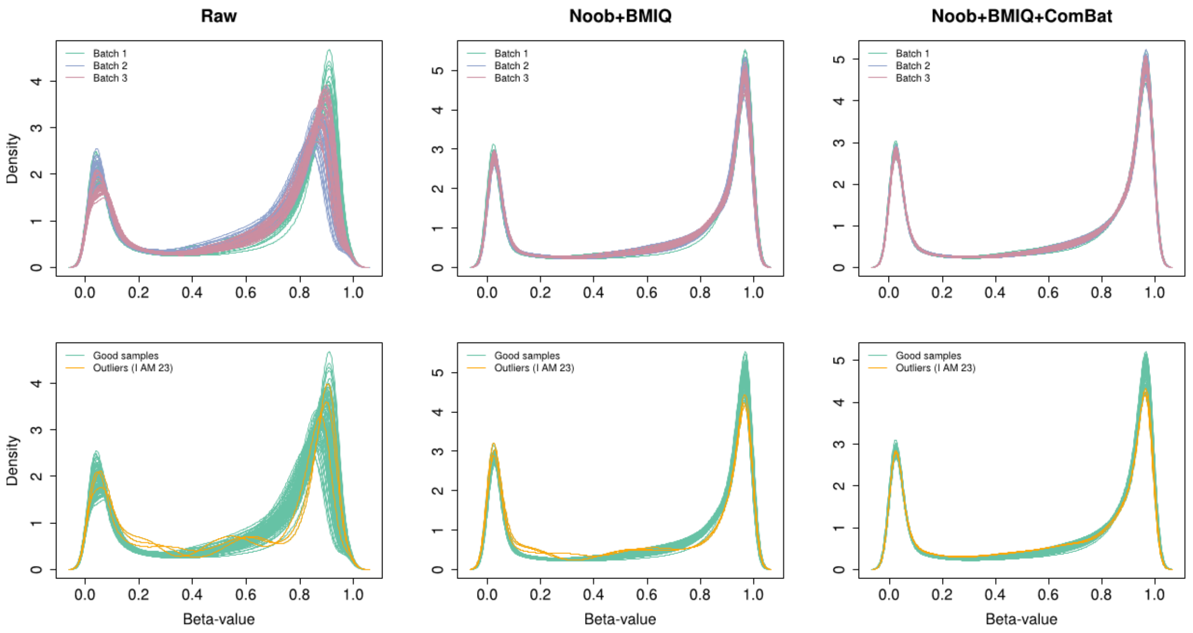

Supplementary Figure 1*. Density plots of the global distributions before and after normalization. Batch effects and effects of outliers are removed after performing Noob, BMIQ, and ComBat normalizations.*

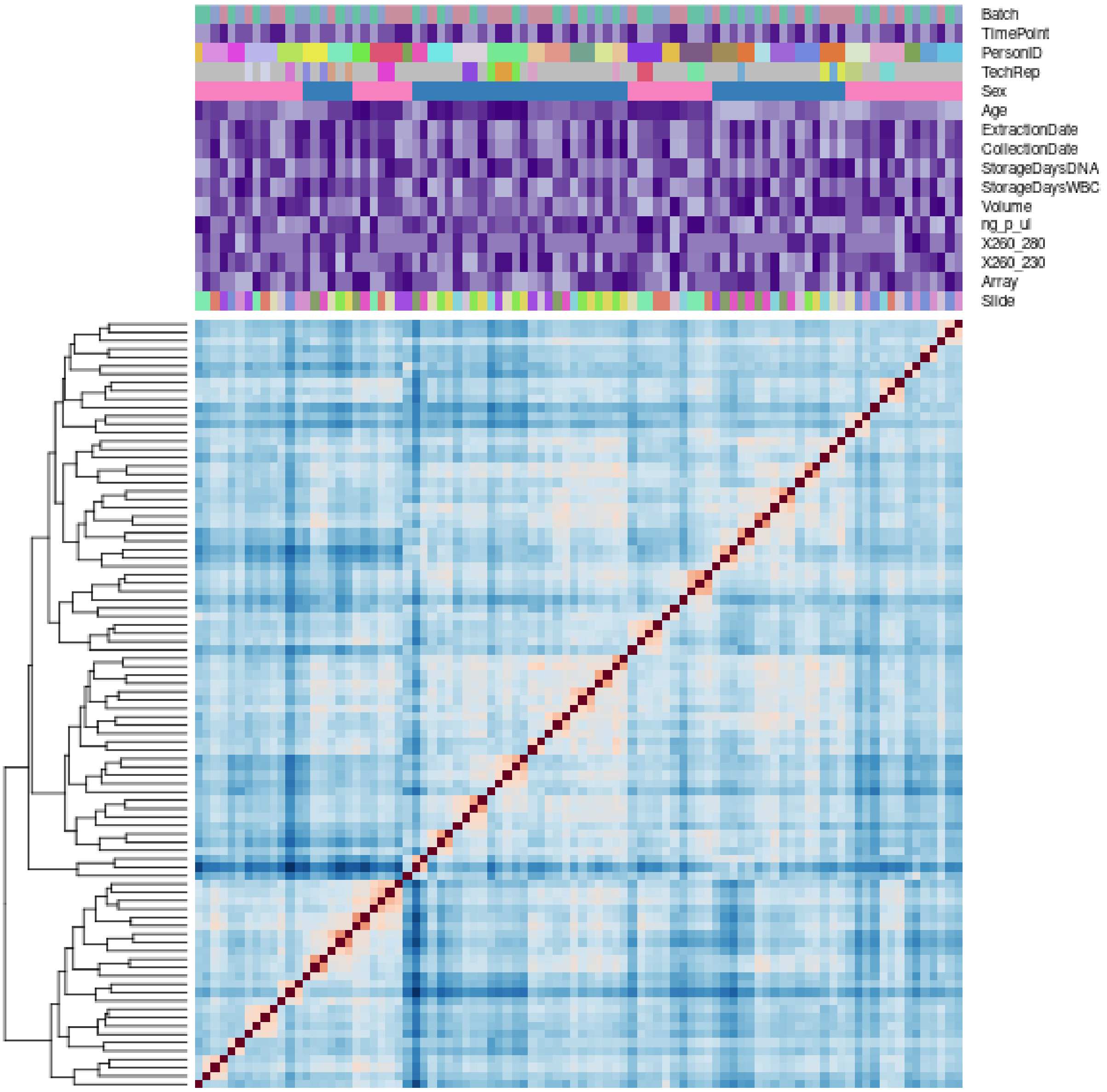

***Supplementary Figure 2**. Unsupervised hierarchical clustering after preprocessing. The preprocessing pipeline successfully removed batch effects; samples of the same person always clustered together.*

2. Disease risk scores, proteomics profiles, physical conditions and blood count predictions

a)

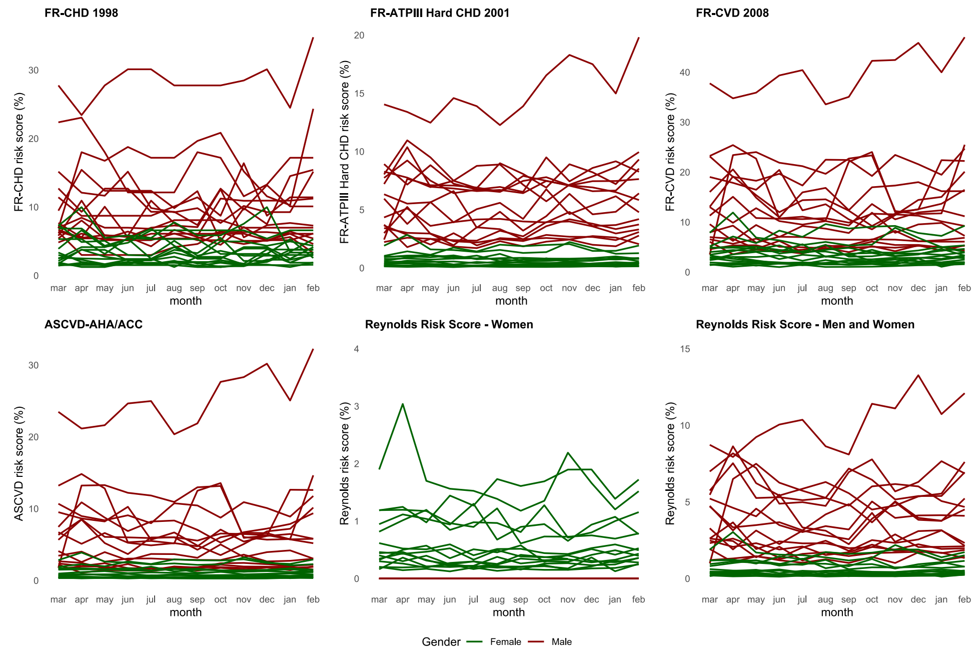

b)

	ID ↓ \ month →	1	2	3	4	5	6	7	8	9	10	11	12
male	01	+	+	+	+	+	+	+	+	+	+	+	+
	02	-	-	-	-	-	-	-	-	-	-	-	-
	03	-	+	-	-	-	-	-	-	-	-	-	-
	04	-	-	-	-	-	-	-	-	-	-	-	-
	05	-	-	-	-	-	-	-	-	-	-	-	-
	06	+	+	+	+	+	+	+	+	+	+	+	+
	07	-	-	-	-	-	-	-	-	-	-	-	-
	08	+	+	+	-	-	-	+	+	-	-	+	+
	09	+	+	+	+	+	+	+	+	+	+	+	+
	10	-	+	+	+	+	+	+	+	-	-	-	+
	11	+	+	+	+	+	+	+	-	-	+	+	+
	12	-	-	-	-	-	-	-	-	-	-	-	-
	13	-	-	-	-	-	-	-	-	-	-	-	-
	14	-	-	-	-	-	-	-	-	-	-	-	-
	15	-	-	-	-	-	-	-	-	-	-	-	-
female	16	-	-	-	-	-	-	-	-	-	-	-	-
	17	-	-	-	-	-	-	-	-	-	-	-	-
	18	-	-	-	-	-	-	-	-	-	-	-	-
	19	-	-	-	-	-	-	-	-	-	-	-	-
	20	-	-	-	-	-	-	-	-	-	-	-	-
	21	-	-	-	-	-	-	-	-	-	-	-	-
	22	-	-	-	-	-	-	-	-	-	-	-	-
	23	-	-	-	-	-	-	-	-	-	-	-	-
	24	-	-	-	-	-	-	-	-	-	-	-	-
	25	-	-	-	-	-	-	-	-	-	NA	-	NA
	26	-	-	-	-	-	-	-	-	-	-	-	-
	27	-	-	-	-	-	-	-	-	-	-	-	-
	28	-	-	-	-	-	-	-	-	-	-	-	-
	29	-	-	-	-	-	-	-	-	-	-	-	-
	30	-	-	-	-	-	-	-	-	-	-	-	-

+ high risk, should be formally screened for diabetes

- low risk

***Supplementary Figure 3**. CVD and diabetes risk scores based on clinical and physiological data. In every time point during the complete 13-month study period, we computed **a**) 10-year cardiovascular disease (CVD) risk scores[7–12], and **b**) Diabetes risk scores (calculated based on the American Diabetes Association (ADA) risk calculator[13].*

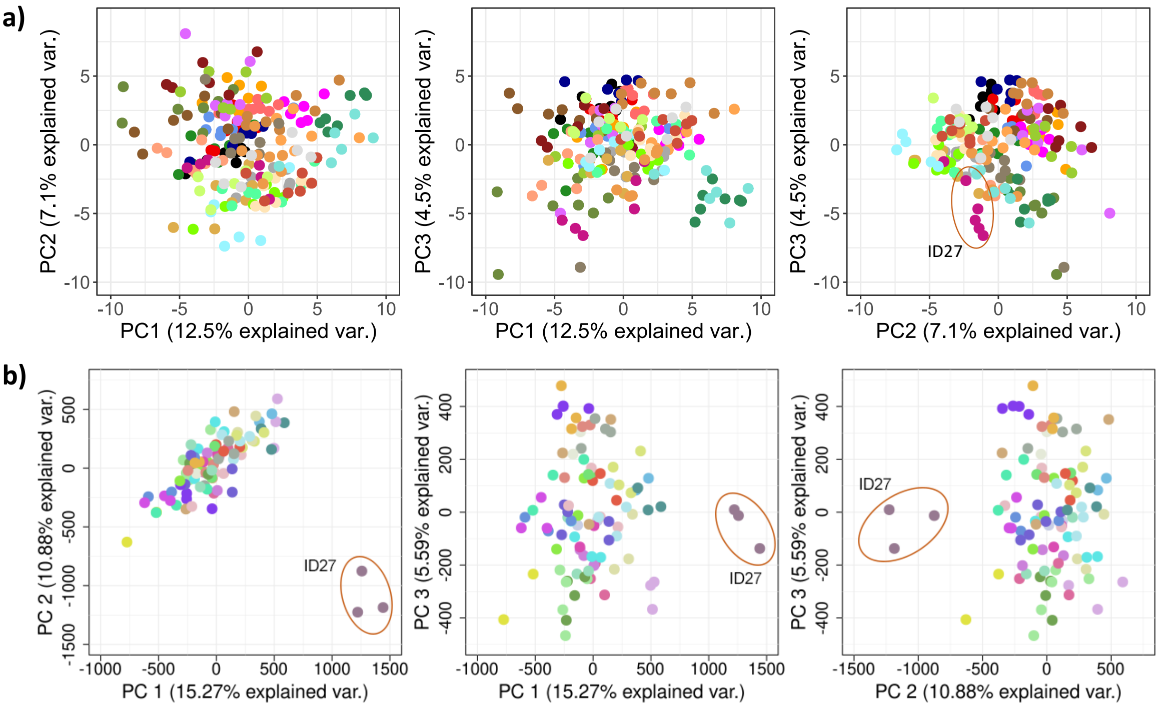

***Supplementary Figure 4**. Unsupervised principal component analysis of proteomics and DNA methylation data. Each color represents different subjects at all available time points. **a)** Principal component analysis of the proteomics dataset. The explained variabilities were calculated using 1067 protein variables in seven time points. ID27 is found outside the main cluster based on the third principal component. **b)** Principal component analysis of the preprocessed, normalized and batch effect-corrected DNA methylation dataset containing 836,477 CpGs of 30 individuals across three time points as well as technical replicates (n=95 samples). All samples of ID27 are located outside the main cluster in principal components one and two.*

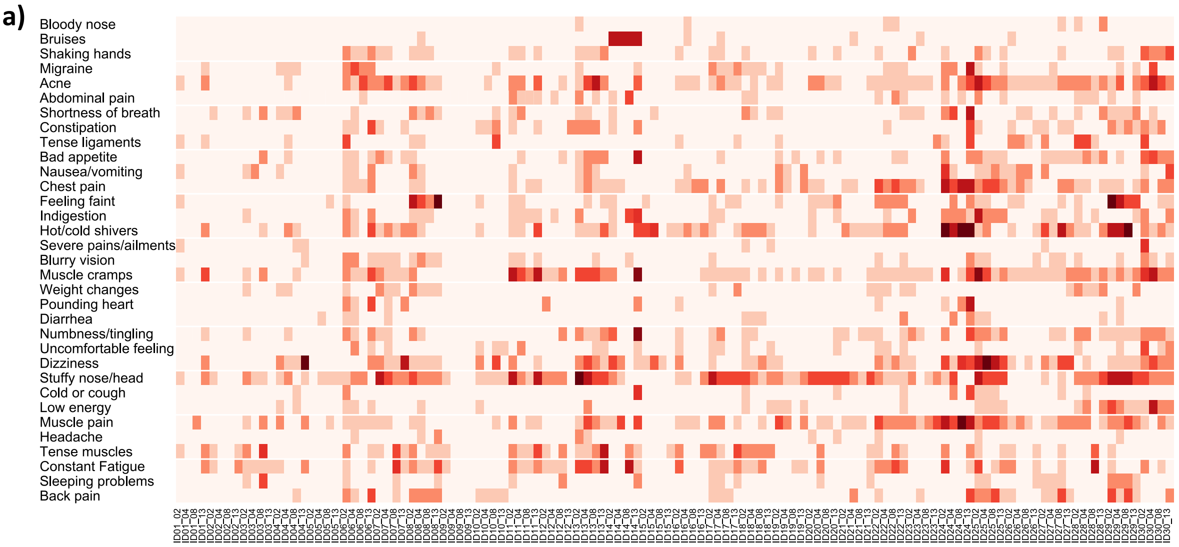

b)

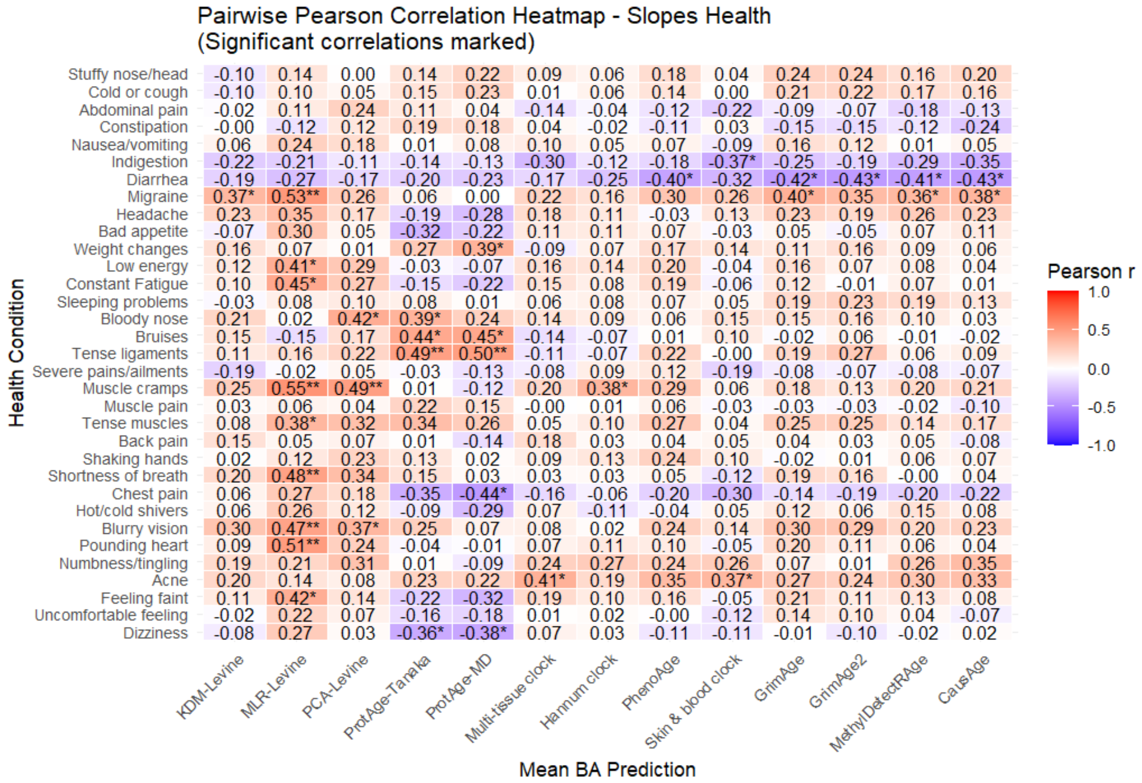

***Supplementary Figure 5**. **a)** Heatmap of the physical condition. Darker colors indicate that individuals experienced the condition more often. The numbers after the identifiers indicate the month that matched with the DNA methylation time points; month 2, 4, 8, and 13. **b)** Pairwise correlations between health trends, measured as slopes of the health complaints over one year, and the average biological age (BA) predictions from each clock. The data were collected based on weekly questionnaire answers.*

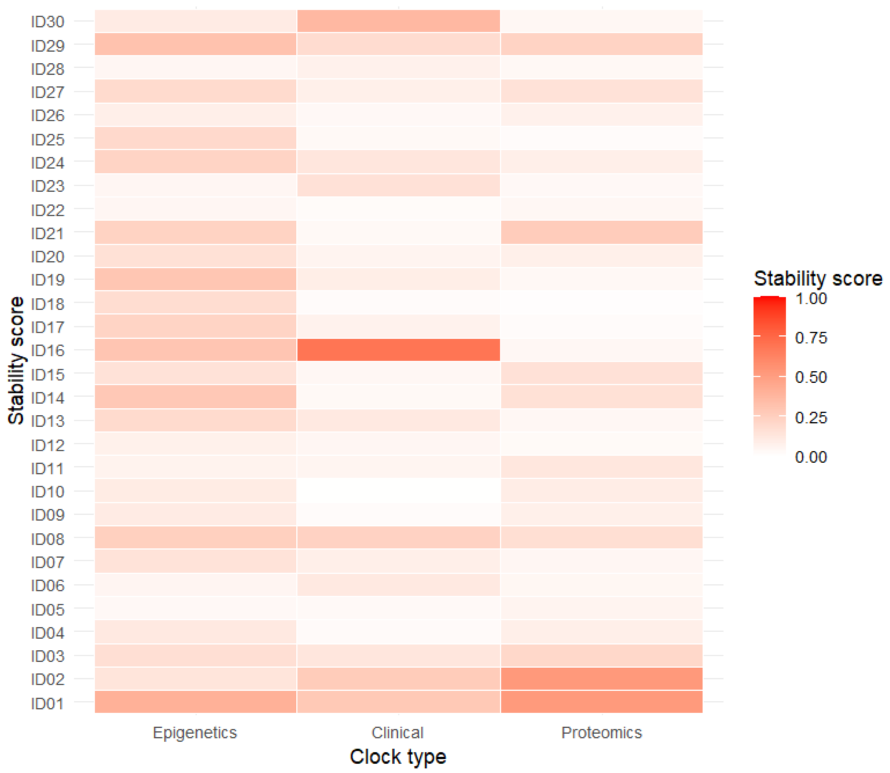

Supplementary Figure 6. *Heatmap showing stability scores (normalised to 0–1) for each individual across epigenetic, clinical, and proteomic clock types. A few individuals exhibit distinctly higher stability in the clinical and proteomic clocks, suggesting greater within-person variability over time. In contrast, epigenetic clocks tend to produce more consistent predictions across individuals.*

a) Blood count prediction - Minfi

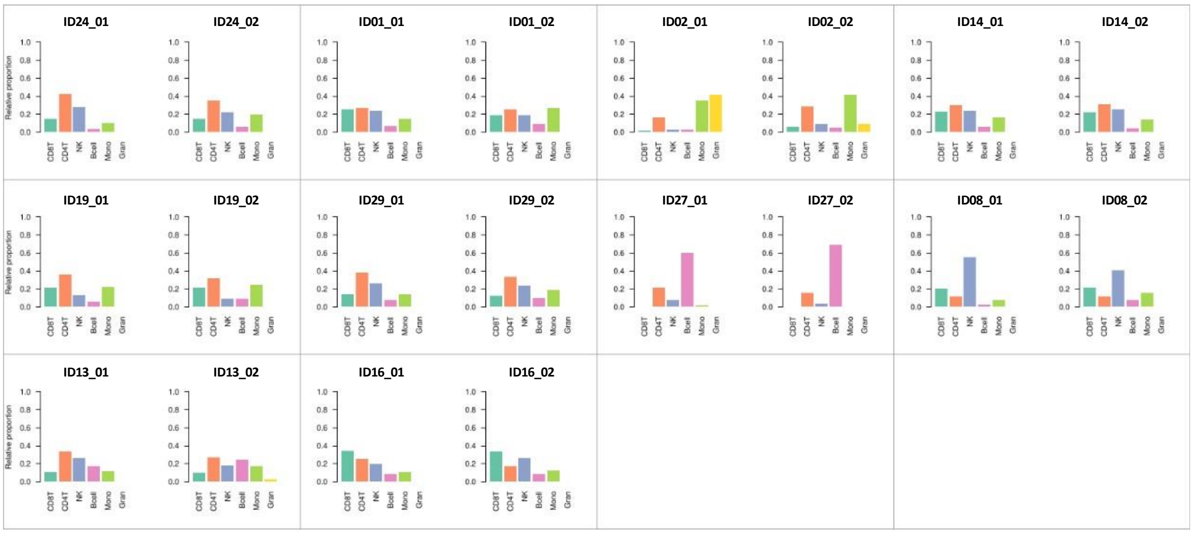

b) Blood count prediction - IDOL

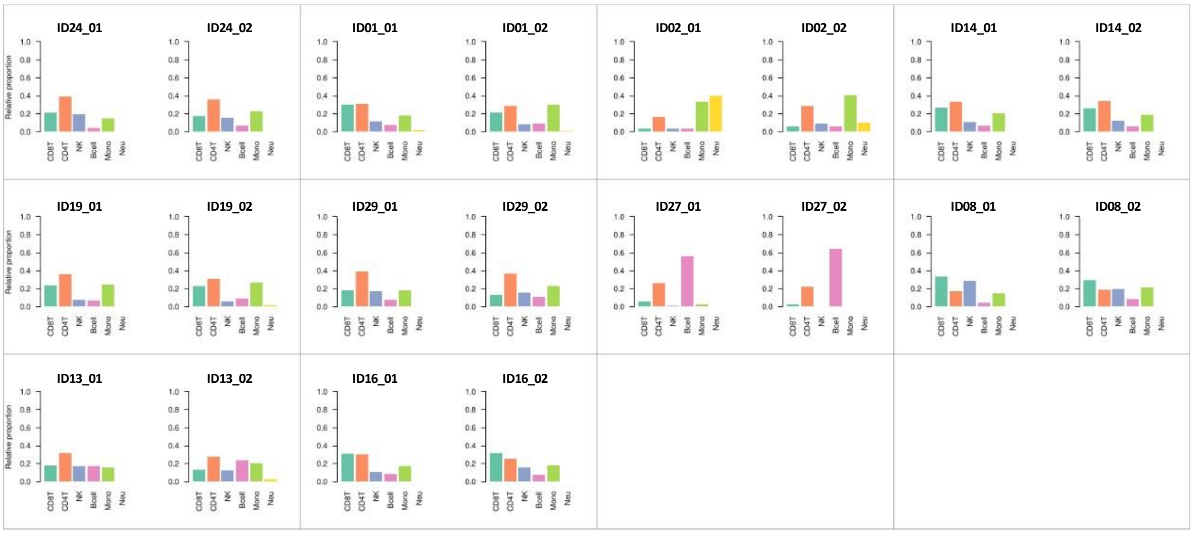

Supplementary Figure 7. *Blood count predictions. The predictions were estimated by* ***a)*** *Minfi and* ***b)*** *IDOL in several individuals. A strong increased proportion of B-cell is observed on ID27, indicating a hematological problem.*

Supplementary References

1. Aryee, M. J. *et al.* Minfi: a flexible and comprehensive Bioconductor package for the analysis of Infinium DNA methylation microarrays. *Bioinformatics* **30**, 1363–1369 (2014).
2. Xu, Z., Niu, L., Li, L. & Taylor, J. A. ENmix: a novel background correction method for Illumina HumanMethylation450 BeadChip. *Nucleic Acids Res* **44**, e20–e20 (2016).
3. Heiss, J. A. & Just, A. C. Identifying mislabeled and contaminated DNA methylation microarray data: an extended quality control toolset with examples from GEO. *Clin Epigenetics* **10**, 73 (2018).
4. Triche, T. J., Weisenberger, D. J., van den Berg, D., Laird, P. W. & Siegmund, K. D. Low-level processing of Illumina Infinium DNA Methylation BeadArrays. *Nucleic Acids Res* **41**, e90–e90 (2013).
5. Teschendorff, A. E. *et al.* A beta-mixture quantile normalization method for correcting probe design bias in Illumina Infinium 450 k DNA methylation data. *Bioinformatics* **29**, 189–196 (2013).
6. Leek, J. T., Johnson, W. E., Parker, H. S., Jaffe, A. E. & Storey, J. D. The sva package for removing batch effects and other unwanted variation in high-throughput experiments. *Bioinformatics* **28**, 882–883 (2012).
7. Wilson, P. W. F. *et al.* Prediction of Coronary Heart Disease Using Risk Factor Categories. *Circulation* **97**, 1837–1847 (1998).
8. Expert Panel on Detection, E. and T. of H. B. C. in A. Executive Summary of the Third Report of the National Cholesterol Education Program (NCEP) Expert Panel on Detection, Evaluation, and Treatment of High Blood Cholesterol in Adults (Adult Treatment Panel III). *JAMA: The Journal of the American Medical Association* **285**, 2486–2497 (2001).
9. D'Agostino, R. B. *et al.* General Cardiovascular Risk Profile for Use in Primary Care. *Circulation* **117**, 743–753 (2008).
10. Goff, D. C. *et al.* 2013 ACC/AHA Guideline on the Assessment of Cardiovascular Risk. *Circulation* **129**, (2014).
11. Ridker, P. M., Buring, J. E., Rifai, N. & Cook, N. R. Development and Validation of Improved Algorithms for the Assessment of Global Cardiovascular Risk in Women. *JAMA* **297**, 611 (2007).
12. Ridker, P. M., Paynter, N. P., Rifai, N., Gaziano, J. M. & Cook, N. R. C-Reactive Protein and Parental History Improve Global Cardiovascular Risk Prediction. *Circulation* **118**, 2243–2251 (2008).
13. Bang, H. Development and Validation of a Patient Self-assessment Score for Diabetes Risk. *Ann Intern Med* **151**, 775 (2009).
